# Faith, food, and healing: a global cross-sectional study of knowledge, attitudes, and practices related to Islamic complementary and alternative foods and remedies

**DOI:** 10.1186/s12906-026-05506-z

**Published:** 2026-08-13

**Authors:** MoezAlIslam E. Faris, Dana N. Abdelrahim, Alyaa Masaad, Sadi Taha, Jehad R. Al Bayari, Moien AB. Khan, Fatima Zohra M Bettayeb, Kabiru Goje, Huthaifah AlKhatib, Howeida Abusalih, Refat Alkurd, Ahmad Sundookah, Husam Khraiwesh, Basil H. Aboul-Enein

**Affiliations:** 1 Department of Clinical Nutrition and Dietetics, Faculty of Allied Medical Sciences, Applied Science Private University, Amman, Jordan; 2 Research Institute for Medical and Health Sciences, College of Health Sciences, The University of Sharjah, Sharjah, UAE; 3 Department of Clinical Nutrition and Dietetics, College of Health Sciences, University of Sharjah, Sharjah, UAE; 4 Faculty of Hotel Management/Events Business, Ammon Applied University College, Amman, Jordan; 5 Department of Business Management, Faculty of Business and Law, The British University in Dubai, Dubai, UAE; 6 Health and Wellness Research Group, Department of Family Medicine, College of Medicine and Health Sciences, United Arab Emirates University, Al-Ain, UAE; 7 Research Institute of Humanities and Social Sciences, University of Sharjah, Sharjah, United Arab Emirates; 8 College of Sharia and Islamic Studies, University of Sharjah, Sharjah, United Arab Emirates; 9 Department of Islamic Sciences, Faculty of Islamic Sciences, Mardin Artuklu University, Mardin, Turkey; 10 Department of Health Sciences, College of Health and Rehabilitation Sciences, Princess Nourah Bint Abdulrahman University, P.O. Box 84428, Riyadh, Saudi Arabia; 11 Department of Nutrition, Faculty of Pharmacy and Medical Sciences, The University of Petra, Amman, Jordan; 12 Department of Nutrition and Food Processing, Faculty of Agricultural Technology, Al-Balqa Applied University, Salt, Jordan; 13 London School of Hygiene & Tropical Medicine, Faculty of Public Health and Policy, Tavistock Place, London, WC1H 9SH UK; 14 College of Arts & Sciences Health & Society Program, University of Massachusetts Dartmouth, 285 Old Westport Rd, North Dartmouth, MA 02747 USA

**Keywords:** Prophetic Medicine, Alternative Treatments, Religious Beliefs, Social media, Evidence-Based Medicine

## Abstract

The increasing interest in alternative and complementary treatments—particularly those referenced in Islamic sources, such as the Holy Qur’an and the Prophetic Sunnah (HQPS) — has led to their growing use as alternatives or adjuncts to modern evidence-based medicine. This global cross-sectional study examines the knowledge, attitudes, and practices (KAP) of adult Muslims regarding the foods and remedies mentioned in the HQPS, along with their associated factors and determinants. An electronic questionnaire comprising twenty-seven questions was developed and verified for clarity. The study included 2,292 participants with a mean age of 38.7 ± 12.7 years, comprising 60.7% females, from 32 countries. The study results indicated adequate knowledge (80.8%; 4.8/6.0), positive attitudes (94.7%; 3.75/4.0), and adherence to Islamic practices regarding the foods and remedies mentioned in the HQPS. The knowledge score was negatively correlated with age (*r* = -0.066, *p* = 0.001) and the practice score (*r* = -0.133, *p* < 0.001), whereas it was positively correlated with the attitude score (*r* = 0.042, *p* = 0.043). Females (*p* < 0.001), employees (*p* = 0.041), and those having a bachelor’s degree (*p* < 0.001) demonstrated better knowledge. The Structural Equation Modeling test revealed that religious commitment, introduced as a moderator, has a significant positive impact on practices, thereby bridging the gap between attitude and practice. Most participants reported frequently adhering to Islamic practices. The study recommends formalizing the therapies outlined in the HQPS and deepening sound scientific knowledge that integrates Islamic principles for preserving the body with modern evidence-based medical practices.

## Introduction

The demand for alternative and complementary treatments, including medicinal herbs and traditional and cultural remedies, has grown significantly in recent decades despite the availability of modern medical services [1]. The rising costs of pharmaceuticals, contemporary treatments, and health insurance [2] have driven many to prefer less expensive, more accessible traditional and folk treatments [3], even among residents of some high-income countries [2, 4, 5]. Foods and natural remedies (e.g., honey, palm dates, garlic, *Zamzam* water (Holy, sacred, healing water from Mecca), black seed mentioned in the Holy Qur’an and the Prophetic Sunnah (HQPS) (the teachings and practices of the Prophet Muhammad, Peace Be Upon Him), and serve as an authentic secondary source of Islam after the Holy Qur’an, are some of the most prominent examples of treatments used in complementary and alternative medicine (CAM), particularly due to their spiritual/religious significance values and medicinal properties. These practices have been described as the traditional Arabic and Islamic medicine (TAIM) model, consisting of an empirical basis for three elements: spiritual healing, herbal medicine, and dietary practices [6].

Given their religious significance, speculation about their effectiveness in comparison to modern medical treatments has increased [7]. Claims of no harmful effects, regardless of the patient’s health status, have become more prevalent. Recent research has confirmed the effectiveness of certain foods and treatments in addressing specific diseases. For example, honey has been found to treat certain burns and sores due to its immunostimulatory action, which promotes autolytic debridement and accelerates the healing of wounded tissues and dormant wounds, in addition to its antimicrobial properties [8]. Similarly, black seeds enhance immunity and improve resistance to chronic and infectious diseases due to their high thymoquinone content. This potent bioactive compound mitigates oxidative stress and restores the balance between pro- and anti-inflammatory cytokines [9]. Combining black seeds with honey and olive oil has been shown to enhance the phenolic and flavonoid content, which are critical contributors to antioxidant activity [10]. Cupping therapy (*Hijama*) has shown promise in treating certain medical conditions [11, 12]. However, many of these treatments remain under investigation and require standardization within the medical field for further controlled clinical testing.

Additionally, research indicates that some of these foods and remedies may have adverse side effects when used without moderation or without considering the patient’s health condition and coexisting pathologies [13, 14], such as the adverse effects of cupping therapy on heart disease, the excessive intake of dates and honey by diabetic patients, or the adverse effects of black seed oil on individuals with liver disease. The widespread claims and speculations about these remedies on social media platforms have significantly influenced consumers’ attitudes, beliefs, and practices [15, 16], a matter that warrants further investigation.

In Islam, the sacred texts contain numerous preventive health teachings and instructions [17] that contribute to improving individual and community health, as well as preventing and reducing the risk of various communicable and non-communicable diseases [18] such as obesity [19–21], infections [22], oral diseases [23], sexually transmitted diseases [24, 25], and the harmful effects of alcohol, which have extensive health, environmental, social, and economic consequences [26–28]. These religious teachings also address social and psychological issues [29, 30], as well as environmental pollution [31, 32].

These religious and doctrinal factors can significantly influence health behaviours and practices related to illness and wellness [33]. Therefore, it is crucial to study Muslim knowledge, attitudes, and practices regarding these foods and remedies, as well as the factors influencing them, using the KAP approach. This evaluation will enable a scientific and medical assessment of the level of awareness. KAP model studies consider that adequate knowledge promotes positive attitudes, which in turn influence promising practices [34]. The results about how these elements relate to one another, however, have been inconsistent. A study conducted by El Haddad et al. found that knowledge did not significantly affect practices [35]. In contrast, another study by Taha et al. found that knowledge had an impact on attitude [36], while Baser et al. [37] found that attitude partially mediates the relationship between knowledge and practice, as noted by Al Bayari et al. [38] found that religious commitment influences practices. Persynaki et al. concluded that religious commitment influences health behaviors, including preventive care attendance, improving diet, exercising, drinking water, and participating in activities that contribute to improved health status [39]. Krause et al. also noted that religious commitment has a positive impact on mental health, including a decrease in stress and an improvement in self-esteem and overall well-being [40].

Although several studies have examined the prevalence of using foods and remedies mentioned in the HQPS among various Muslim groups [7, 19, 41, 42]No one has explored these remedies from the perspective of modern medical practice or their applications in contemporary healthcare systems. Furthermore, no studies have comprehensively investigated the views, attitudes, and practices of adult Muslims toward these foods and treatments mentioned in the HQPS or scientifically evaluated these beliefs.

**Fig. 1:**
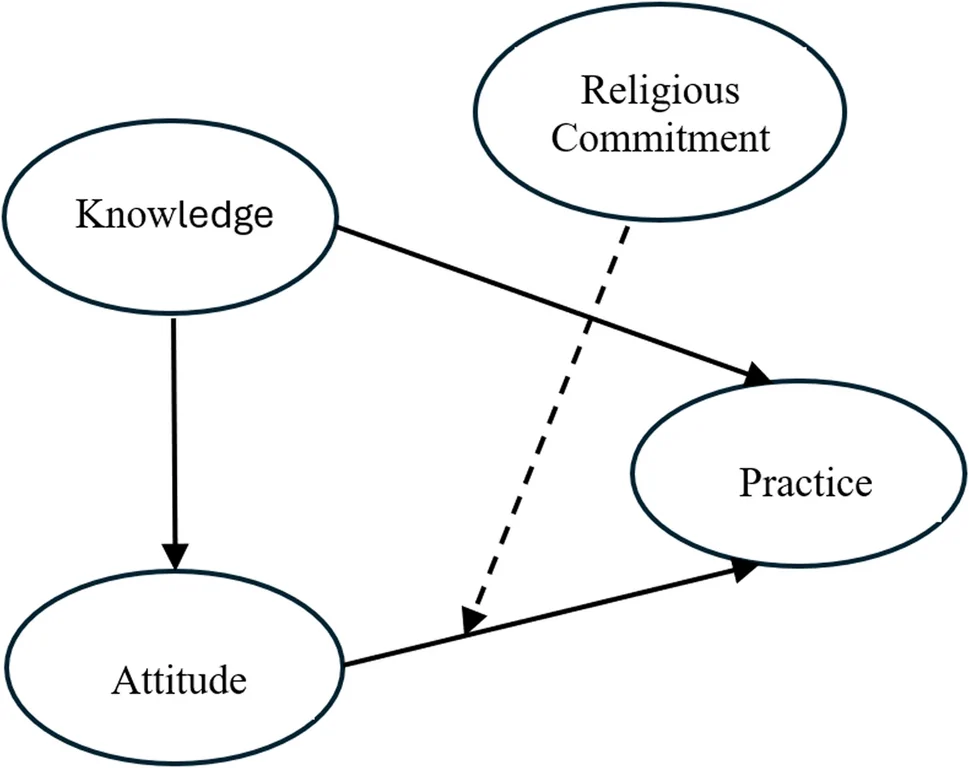
Proposed conceptual model. (*n* = 2,292)

Therefore, this cross-sectional study was designed to explore the KAP of adult Muslims regarding natural foods and remedies mentioned in the HQPS. It focused on how these individuals integrate nutrition and medicine with their religious beliefs, and on the specific practices they follow when using these foods and remedies to maintain health and mitigate disease. The relationships proposed in this study are presented in the conceptual model (Fig. 1).

## Materials and methods

### Ethical issues

Ethical approval was obtained from the Research Ethics Committee (REC) at the University of Sharjah (REC-24-05-22-01-PG). The completion of the questionnaire was considered a form of signed consent by each participant, in accordance with the ethical principles of scientific research and the Universal Declaration of Helsinki. Participants’ confidentiality was confirmed, and the study’s academic objectives were clearly stated and distinguished from any advertisement.

### Sample size and sampling

Web-based questionnaires were distributed, and participants were recruited through convenience and snowball sampling. The sample size was based on prior studies [43] and on practical considerations regarding time, funding, and workforce that play a role in determining the sample size, while balancing the need for statistical rigor with available resources. The minimum final sample size required for your Global KAP study, considering a 95% confidence level, a 5% margin of error, design complexity, and non-response, is 722 participants. This minimum number of participants is recommended for survey research to enable reliable subgroup analyses.

### Questionnaire validation

The questionnaire was structured as a self-administered instrument and was translated and pre-tested to confirm clarity and avoid ambiguity. A pilot study was conducted with 15 participants who met the inclusion criteria to ensure the clarity and validity of the questions and verify their alignment with scientific evidence and Islamic rulings, thereby avoiding any potential ethical violations or religious conflicts. Since no revisions to the questionnaire were needed after pilot testing, the pilot participants were included in the final analysis sample. The questionnaire developed for this research was translated into English. The translation and cultural adaptation process adhered to the “Principles of Good Practice for the Translation and Cultural Adaptation Process.” [44]. Initially, forward translations were carried out by two independent translators, both fluent in Arabic and English, and were then back-translated into Arabic. The backward translation was reviewed, and the questionnaire versions for each language were proofread and further refined as needed. These versions were then pilot-tested again with 35 participants, leading to the finalized questionnaires [44]. The questionnaire was reviewed by experts in Islamic studies, education, psychology, dietetics, and epidemiology, and subsequently adapted to an electronic format in Google Forms, which is available in both languages. Participants were recruited via social media platforms, including Facebook, WhatsApp, X, and Instagram, to reach a geographically diverse sample across multiple countries efficiently. The questionnaire had an approximate completion rate of 60–70%, reflecting participants’ engagement.

### Participants’ inclusion and exclusion criteria, data settings

Participants were recruited from 32 countries distributed over four continents (Asia, Africa, Europe and North America): Jordan, Palestine, Syria, Saudi Arabia, United Arab Emirates, Bahrain, Oman, Kuwait, Egypt, Iraq, Lebanon, Yemen, Libya, Tunisia, Algeria, Morocco, Sudan, Qatar, Turkey, Iran, India, Malaysia, Somalia, Nigeria, Ghana, Senegal, Djibouti, Central African Republic, Canada, United States of America, United Kingdom, and Germany”, via web-based questionnaires distributed through social media platforms, online forums, and professional and religious networks. Strict criteria were used to define the sample for this global cross-sectional study to ensure the relevance and validity of the collected KAP data. Participants must be 18 years of age or older, self-identify as Muslim, and be able to read and understand one of the languages in which the survey is disseminated (e.g., English, Arabic). Furthermore, participants were required to provide explicit online consent before commencing the questionnaire, have reliable internet access, and a suitable device for completion. Conversely, participants were excluded if they were under 18 years of age, non-Muslim, failed to provide informed consent, exhibited poor data quality (e.g., inconsistent response patterns), had cognitive or communication limitations that prevented completion of the survey, or failed to complete a critical portion of the survey. Regarding educational level, participants must have at least a basic level of education, defined as completion of primary/elementary education, to adequately understand and respond to the survey. This criterion enables us to examine how levels of education influence KAP regarding Islamic complementary and alternative foods and remedies. Key persons in various countries were contacted to facilitate the dissemination of the electronic questionnaire among Muslim communities through their societies, organizations, and gatherings, as well as via social media platforms specifically designed for Muslim online communities worldwide. Data were collected over an extended period, from November 2019 to October 2024, to allow sufficient time to recruit and enroll a large, diverse sample.

### Data collection tool

Participants were asked to describe their knowledge and attitudes regarding HQPS foods and remedies using an online, self-administered questionnaire. The questionnaire consisted of 27 closed-ended questions distributed over five domains. This questionnaire has been evaluated for validity and reliability, with a Cronbach’s alpha of 0.85, indicating acceptable internal consistency. It consistently measures KAP related to foods and remedies from the HQPS. Participants were allowed to complete the questionnaire at their own pace, typically taking 15–25 min, to ensure thoughtful and unhurried responses.

The first domain comprised eight sociodemographic questions: sex, age, educational level, occupation, marital status, economic status, self-assessments of adherence to and commitment to religion, and nationality. The second domain assessed the sources of information on the foods and remedies mentioned in the HQPS using multiple-choice questions. The third domain comprises six questions that evaluate beliefs and knowledge regarding the foods and remedies mentioned in the HQPS. The fourth domain included four questions concerning attitudes towards the foods and remedies mentioned in the HQPS for the medical treatment of common diseases and ailments. The latter two domains used a 5-point Likert scale, ranging from “Strongly Disagree” to “Strongly Agree.” The fifth domain assessed personal practices related to the foods and remedies mentioned in the HQPS. This section used five questions on a 5-point Likert scale, ranging from “never” to “always.” This final section also includes two multiple-choice questions to determine the foods and remedies associated with these practices. Religious commitment was assessed with a single self-report item in which participants rated their level of commitment as low, moderate, or high as per the Religious Commitment Scale [45–47]. Low religious commitment refers to individuals who identify as Muslim but engage minimally with their faith, attending religious activities infrequently and having limited participation in community events. Moderate commitment captures individuals who actively participate in their faith through regular attendance at religious activities and a moderate understanding of relevant practices. Lastly, high religious commitment refers to highly engaged individuals who regularly attend religious activities and deeply integrate religious teachings and practices into their daily lives.

The foods referenced in the HQPS for this study were obtained from the book *“Food in the Holy Qur’an from the Perspective of Modern Nutrition*,*”* published by the University of Hail, Kingdom of Saudi Arabia, in 2015 [48]. Moreover, the foods and treatments referenced in the Prophetic *Sunnah* were collected from the doctoral dissertation on the principles of Islamic jurisprudence, *“Prophetic Drug Treatments in Light of Modern Pharmacological Studies - A Comparative Nodalism Study.”* [49].

### Statistical analysis

Socio-demographic data, including sex, age, educational level, occupation, marital status, self-assessed economic status, level of adherence to or commitment to religion, and nationality, were presented as categorical variables using frequencies and percentages. Continuous variables, such as age, were reported as mean ± standard deviation (SD). The total scores for knowledge, attitude, and practice were treated as continuous variables and reported as mean ± SD. Categorical descriptions, including frequencies and percentages, were also provided for the variables, which were categorized using established cutoff points, yielding binary classifications based on each variable’s mean score. A correlation test was conducted to assess the relationship between two continuous variables. Cross-tabulations and Chi-Square tests were used to determine associations between categorical variables. Data analysis was performed using IBM SPSS Version 29.0 (USA) and AMOS (Maximum Likelihood estimation), with a significance level set at *P* < 0.05. Inspection of descriptive statistics and inter-item correlations revealed no major departures from normality or multicollinearity, supporting the suitability of the data for SEM analysis using AMOS.

## Results

### Descriptive statistics

Out of the 2,292 participants who completed the questionnaire, 1392 (60.7%) were females. The mean age of participants was 38.7 ± 12.7 years, with an age range of 18–80 years. More than half had a bachelor’s degree (57%), and one-third (33.6%) had a post-graduate degree. More than half (55.1%) were employed, 14.5% were homemakers, 13.8% were school or university students, and 6.9% were unemployed. Two-thirds of the participants were married (66.6%), followed by single participants (29.1%). Regarding economic status, 78% of participants described themselves as middle class, 9.9% as high class, and the least (2.4%) as from a low class. Among the entire sample, less than two-thirds (63.5%) described themselves as moderately religious, almost one-third (34.1%) as highly religious, and around 2.4% as less religious. When it comes to nationalities, the majority (58.5%) were from the Levant region, 22.7% were from the Arabian Gulf, 9.2% were from the Sub-Saharan African region, 5.7% from Northern Africa, and the remaining 3.8% were distributed among Europe, North America, and Asia, as illustrated in Table 1.

**Table 1:** Sociodemographic characteristics of the study participants (*n* = 2,292)

Characteristics		Frequency	Percentage (%)
Age (Mean ± SD) = 38.7 ± 12.7 range 18 to 80 years			
Sex	Male	900	39.3
	Female	1392	60.7
Educational level	Elementary/primary	13	0.6
	Secondary/high school	204	8.9
	University (B.Sc.)	1306	57.0
	Postgraduate (M.Sc./ Ph.D.)	769	33.6
Job/Occupation	Housewives	332	14.5
	Student	317	13.8
	Work owner	57	2.5
	Employee	1262	55.1
	Retired	165	7.2
	Unemployed	159	6.9
Marital status	Single	667	29.1
	Married	1527	66.6
	Divorced	62	2.7
	Widowed	36	1.6
Self-described economic status	Low	277	12.1
	Medium	1788	78.0
	High	227	9.9
Self-described level of religious commitment	Low	56	2.4
	Moderate	1455	63.5
	High	781	34.1
Country/Nationality	Gulf	521	22.7
	Levant	1341	58.5
	North Africa	131	5.7
	Sub-Saharan Africa	211	9.2
	Europe, North America, and others	25	1.1
	Asia	63	2.7

### Knowledge and beliefs toward foods and remedies of the HQPS

When participants were asked about their religious beliefs regarding the necessity of consuming food and medicine to preserve life and protect the body from harm, 98.4% agreed (76.1% strongly agreed and approximately 22.3% agreed). Regarding their belief that the foods mentioned in the HQPS (e.g., honey, palm dates, garlic, *Zamzam* water, black seed) can augment and support modern medical remedies, 97.9% agreed (71% strongly agreed and 26.9% agreed). When asked whether remedies mentioned in the HQPS could serve as supportive measures for modern medical treatments to address illnesses and prevent ailments, 90.2% agreed (38.6% agreed and 51.6% strongly agreed). Almost half of the participants (50.4%) believed that foods and remedies mentioned in the HQPS could serve as viable alternatives to classical and modern therapies, while around one-third (34.9%) disagreed. Additionally, one-quarter (26.4%) of participants believed that these foods cannot cause harm to the user, regardless of the quantity consumed or the user’s health status. In comparison, about two-thirds (65.3%) disagreed. Similarly, when asked whether these foods or remedies could be applied to all medical conditions without causing harm, the majority (about 63%) disagreed, whereas 27.4% agreed. Participants’ knowledge of the foods and remedies mentioned in the HQPS was good (80.8%). They scored more than 50% by correctly answering at least 4 out of 6 belief-related questions, as shown in Table 2.

**Table 2:** Knowledge of the study participants toward the food and remedies cited in the HQPS (*n* = 2,292)

	Question		Strongly agree	Agree	Neutral	Disagree	Strongly disagree
Q1	I religiously know the necessity of having food and medicine to keep the body and soul safe from damage and harm		1745 (76.1)	511 (22.3)	18 (0.8)	12 (0.5)	6 (0.3)
Q2	I religiously know that having the foods mentioned in the HQPS (e.g., honey, garlic, *Zamzam* water, black seed) is are supportive and strengthening means for modern medical remedies and in treating illnesses and preventing ailments		1627 (71.0)	617 (26.9)	25 (1.1)	17 (0.7)	6 (0.3)
Q3	I firmly believe that it is possible to use the remedies mentioned in the Prophetic Sunnah (such as cupping, cauterization, etc.) to support modern medical treatments for the prevention and treatment of diseases.		1183 (51.6)	885 (38.6)	125(5.5)	87 (3.8)	12 (0.5)
Q4	I know that the foods and remedies mentioned in the HQPS can be viable replacements for traditional and modern treatments, so that they can be dispensed in place of those treatments.		544 (23.7)	613 (26.7)	335(14.6)	642(28.0)	158 (6.9)
Q5	I know, on religious grounds, that the foods mentioned in the HQPS cannot cause harm, regardless of quantity, mode of consumption, or the consumer’s health status.		279 (12.2)	325 (14.2)	193 (8.4)	1042(45.5)	453 (19.8)
Q6	I know that the remedies mentioned in the HQPS can be applied to all medical conditions and will have no harmful effects, regardless of the consumer’s health status.		285 (12.4)	343 (15.0)	215 (9.4)	1015 (44.3)	434 (18.9)

	Question	One mark for whom	Strongly Agree and Agree		Strongly Disagree, Disagree, and Neutral		
	Q1	Strongly Agree and agree	2256 (98.4)		36 (1.6)		
	Q2	Strongly Agree and agree	2244 (97.9)		48 (2.1)		
	Q3	Strongly Agree and agree	2068 (90.2)		224 (9.8)		
	Q4	Strongly Disagree, disagree, and neutral	1157 (50.5)		1135 (49.5)		
	Q5	Strongly Disagree, disagree, and neutral	604 (26.4)		1688 (73.6)		
	Q6	Strongly Disagree, disagree, and neutral	628 (27.4)		1663 (72.6)		
	Total knowledge score (mean 4.8 out of 6)						
	Adequate knowledge (> 50%, mean)		1853 (80.8)				
	Inadequate knowledge (*≤* 50%, mean)		439 (19.2)				

**Fig. 2:**
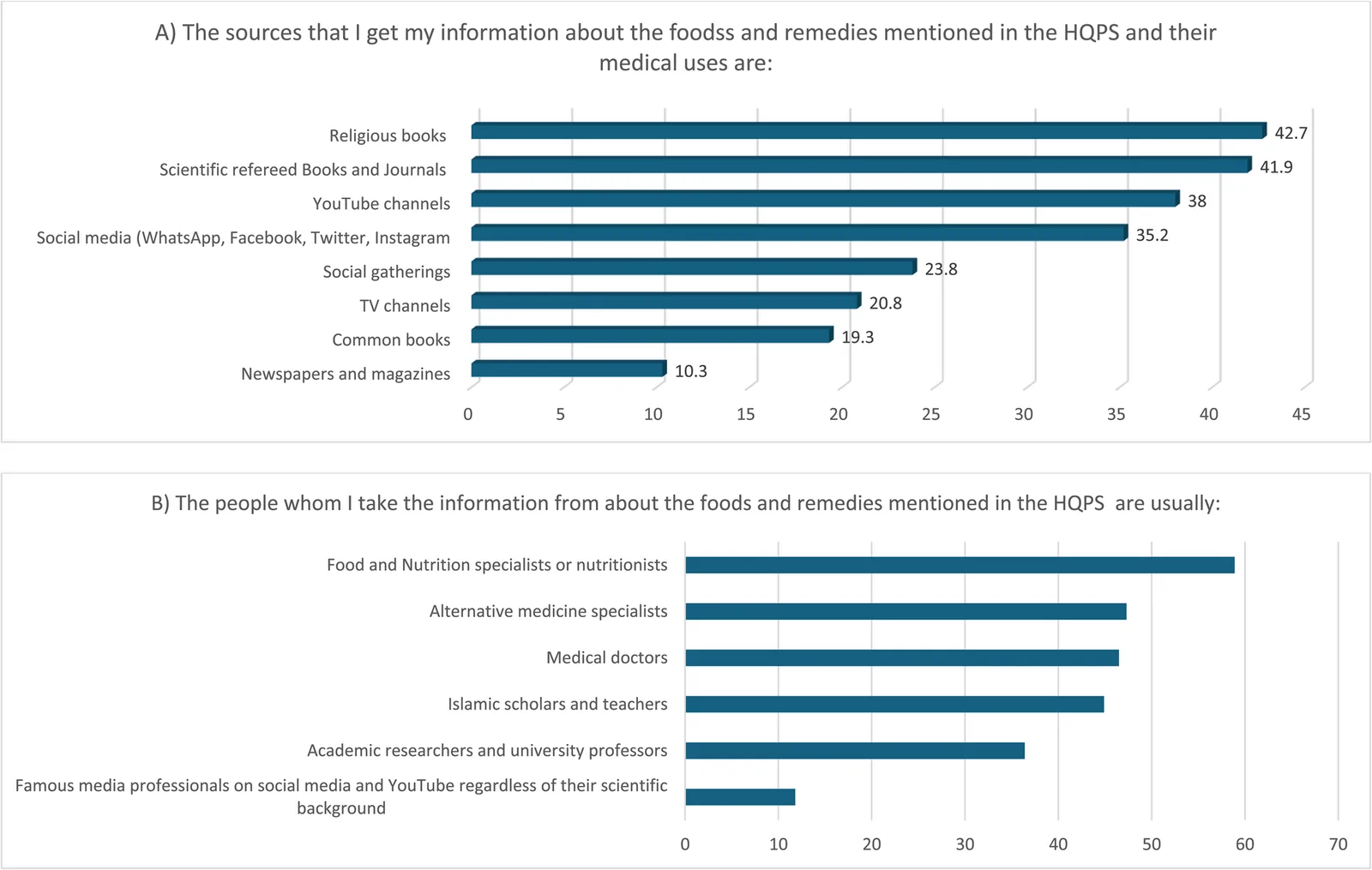
Sources of information from (**A**) resources and (**B**) people reported by the study participants about the foods and remedies cited in the HQPS. (*n* = 2,292)

The most frequently cited sources of information on these foods and remedies were religious books (42.7%), peer-reviewed scientific books and journals (41.9%), YouTube channels (38%), and social media (35.2%). Other sources included social gatherings (23.8%), TV programs (20.8%), non-scientific cultural books (19.3%), and newspapers or cultural magazines (10.3%). In terms of individuals from whom participants received information, nutritionists were the most common source (58.9%), followed by alternative medicine therapists (47.3%), medical doctors (46.5%), Islamic scholars and *Imams* (44.9%), academic researchers (36.4%), and media personalities (11.8%) (Fig. 2).

### Attitudes toward foods and remedies of the HQPS

Table 3 shows participants’ attitudes toward the use of foods and remedies cited in the HQPS. The majority of participants held positive attitudes toward CAM and holistic medicine foods and remedies. Approximately 96.5% agreed that these foods should be promoted as complementary therapies alongside traditional medicine. Similarly, 94% of participants supported the inclusion of these foods and remedies in educational curricula to instill these practices among young people. When asked whether treatments such as cupping therapy and cauterization should be regulated by law and performed by qualified practitioners, 94.1% agreed. Additionally, 90.3% expressed the need for government authorities to monitor and regulate certain practices, such as the use of camel urine, cauterization, cupping therapy, and *Ruqyah*. Overall, 94.7% of participants exhibited a high level of positive attitudes, and more than 50% answered at least 2 of 4 attitude-related questions correctly.

**Table 3:** Attitudes of the study participants toward the food and remedies cited in the HQPS (*n* = 2,292)

No.	Question		Strongly agree	Agree	Neutral	Disagree	Strongly disagree
Q9	I believe it is essential to raise awareness of the use of the foods and remedies mentioned in the HQPS as part of a complementary treatment approach alongside traditional methods.		1407 (61.4)	804 (35.1)	37 (1.6)	36 (1.6)	8 (0.3)
Q10	I believe it is essential to incorporate the foods and remedies mentioned in the HQPS into education curricula to instill in young people a culture of interaction with these traditions.		1275 (55.6)	880 (38.4)	78 (3.4)	47 (2.1)	12 0.5)
Q11	I believe it is essential to legalize the process of administering remedies mentioned in the Prophetic Sunnah (especially cupping and cauterization) so that their practice can be regulated and verified, ensuring that the person administering them is competent and qualified. The health rules and conditions to be followed during its conduct are clearly stipulated.		1460 (63.7)	697 (30.4)	71 (3.1)	48 (2.1)	16 (0.7)
Q12	I think it is important that government sectors monitor some of the medical practices mentioned in the Prophetic Sunnah, like dealing with camel urine, cupping, cauterization, and *Ruqya*		1380 (60.2)	689 (30.1)	117 (5.1)	88 (3.8)	18 (0.8)

	Question	One mark for whom	Strongly Agree and Agree		Strongly Disagree, Disagree, and Neutral		
	Q9	Strongly Agree and Agree	2211 (96.5)		81 (3.5)		
	Q10	Strongly Agree and Agree	2155 (94.0)		137 (6.0)		
	Q11	Strongly Agree and Agree	2157 (94.1)		135 (5.9)		
	Q12	Strongly Agree and Agree	2069 (90.3)		223 (9.7)		
	The total Attitude score (mean 3.75 out of 4)						
	Good attitude (> 50%, mean)		2171 (94.7)				
	Poor attitude (*≤* 50%, mean)		121 (5.3)				

### Practices related to foods and remedies of the HQPS

**Fig. 3:**
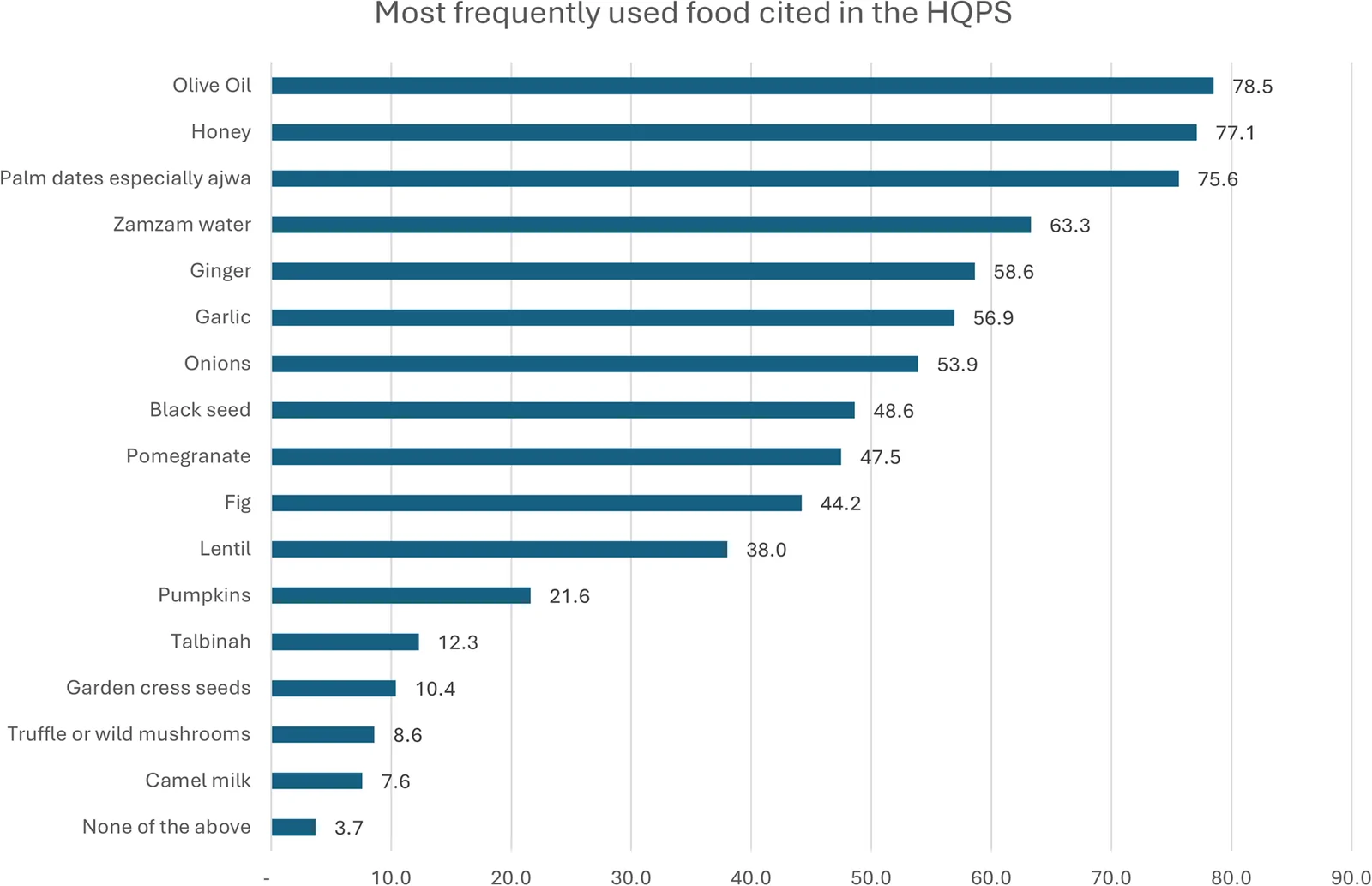
Most frequently used foods cited in the HQPS (Multiple answer question). (*n* = 2,292)

**Fig. 4:**
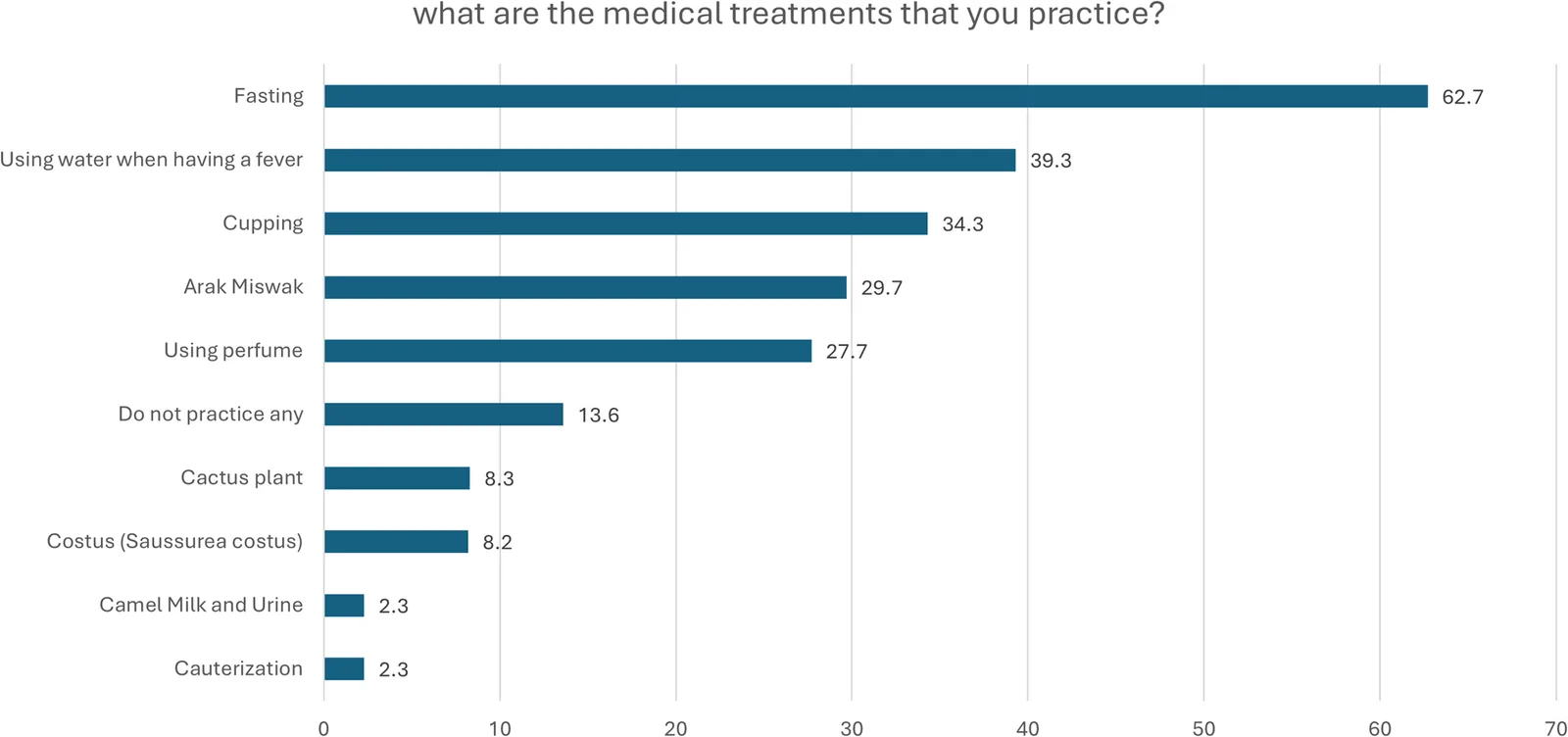
Most frequently used remedies cited in the HQPS (Multiple answer question). (*n* = 2,292)

When participants were asked about their practices related to consuming foods mentioned in the HQPS, 92.8% reported consuming these foods regularly—occasionally, frequently, or consistently—for prevention, healing, or maintaining health, as shown in Table 4. Figure 3 shows the most consumed foods, including olive oil (78.5%), honey (77.1%), palm dates (75.6%), and *Zamzam* water (63.3%). Lesser-consumed items included camel milk, truffles, and garden cress seeds (Fig. 4).

**Table 4:** Practices of the study participants toward the food and remedies cited in the HQPS (*n* = 2,292)

No.	Question		Never	Rarely	Sometimes	Often / Frequently	Always
Q13	I make sure to eat any of the foods and remedies mentioned in the HQPS (like honey, garlic, *Zamzam* water, and black seed) to prevent or cure illnesses or maintain my health *		28 (1.2)	136 (5.9)	756 (33.0)	907 (39.6)	465 (20.3)
Q15	I ensure that I practice any of the medical treatments mentioned in the Prophetic Sunnah (such as cupping, cauterization, or voluntary fasting) to prevent or cure illnesses or to maintain my health.		299 (13.0)	528 (23.0)	803 (35.0)	437 (19.1)	225 (9.8)
Q17	I ensure that I avoid food waste, following Divine and Prophetic guidance.		22 (1.0)	58 (2.5)	418 (18.2)	879 (38.4)	915 (39.9)
Q18	I ensure that I adhere to the manners of eating and drinking as mentioned in the Prophetic Sunnah, such as saying ‘bismillah’ before eating, washing my hands before eating, eating with my right hand and from what is in front of me, and washing my hands and thanking Allah after eating.		3 (0.1)	10 (0.4)	85 (3.7)	559 (24.4)	1635 (71.3)
Q19	I ensure that information about the foods and remedies mentioned in the HQPS, as well as their health-related connections, is credible by consulting reliable scientific sources or specialists, such as physicians or licensed nutritionists.		65 (2.8)	119 (5.2)	357 (15.6)	754 (32.9)	997 (43.5)

	Question	One mark for whom	Never and rarely		Sometimes, Often/ Frequently, and Always		
	Q13	Sometimes, Often/Frequently, and Always	164 (7.2)		2128 (92.8)		
	Q15	Sometimes, Often/Frequently, and Always	827 (36.1)		1465 (63.9)		
	Q17	Sometimes, Often/Frequently, and Always	80 (3.5)		2212 (96.5)		
	Q18	Sometimes, Often/Frequently, and Always	13 (0.6)		2279 (99.4)		
	Q19	Sometimes, Often/Frequently, and Always	184 (8.0)		2108 (92.0)		
		The total practice score (mean 4.44 out of 5)					
	Good practice (> 50%, mean)		1310 (57.2%)				
	Poor practice (*≤* 50%, mean)		982 (42.8%)				

*The item details are shown in Fig. 2

$ The item details are shown in Fig. 3

Regarding the use of prophetic treatments such as cupping therapy, cauterization, or voluntary fasting, 92.8% reported occasional, frequent, or consistent use. Voluntary fasting was the most popular treatment (62.7%), followed by water for fever (39.3%) and cupping therapy (34.3%). Additionally, the majority of participants (96.5%) reported avoiding food waste by adhering to Divine and prophetic guidance. Most participants (99.4%) also adhered to Prophetic etiquette while eating, and 92% regularly verified the credibility of information regarding food and treatments from reliable sources (Table 4). Overall, 57.2% of participants demonstrated a high level of health practices, and more than 50% answered two or more of the five practice-related questions correctly.

Furthermore, the statistical analyses in Table 5 revealed a significant negative correlation between participants’ age and total knowledge score (*r* = -0.066; *p* = 0.001), as well as a positive correlation between knowledge and attitude scores (*r* = 0.042; *p* = 0.043). Additionally, significant positive correlations were observed between age and the total practice score (*r* = 0.162; *p* < 0.001) and between the total attitude score and the total practice score (*r* = 0.101; *p* < 0.001). However, a significant negative correlation was found between the total knowledge score and the total practice score (*r* = -0.113; *p* < 0.001).

**Table 5:** Correlation between age (independent variable) and scores of knowledge and attitude (dependent variables) (*n* = 2,292)

Variable	Age	Knowledge total score	Attitude total score	Practice total score
Age	-	-	-	-
Knowledge total score	-0.066 (0.001)**	-	-	-
Attitude total score	-0.007 (0.755)	0.042 (0.043)*	**-**	**-**
Practice total score	0.162 (<0.001)*	-0.113 (<0.001)*	0.101 (<0.001)*	**-**

* = *p *< 0.01, ** = *p *< 0.001

Table 6 showed that females had better knowledge of the use of foods and remedies mentioned in the HQPS than males (*p* < 0.001). Additionally, participants with a bachelor’s degree (*p* < 0.001) and those who are employed (*p* = 0.041) demonstrated greater knowledge than participants with lower educational degrees, as well as those who are unemployed or otherwise unengaged. Moreover, participants who described their religious commitment as moderate had greater knowledge than those who reported high or low levels of religious commitment (*p* = 0.005). Additionally, participants from the Levant region demonstrated the highest level of knowledge compared to those from other areas (*p* < 0.001).

**Table 6:** Distribution of sociodemographic characteristics based on the knowledge level (*n* = 2,292)

Sociodemographic characteristic		Adequate knowledge	Inadequate knowledge	*P*-value
Sex	Male	681 (36.8)	219 (49.9)	˂0.001**
	Female	1172 (63.2)	220 (50.1)	
Educational level	less than a university	151 (8.1)	66 (15.0)	˂0.001**
	University (BSc)	1089 (58.8)	218 (49.4)	
	Graduate (MSc/ PhD)	613 (33.1)	156 (35.5)	
Job/occupation	Housewife	284 (15.3)	48 (10.9)	0.041*
	Student	252 (13.6)	65 (14.8)	
	Worker/ Employee	1047 (56.5)	272 (62.0)	
	Retired / no work	270 (14.6)	54 (12.3)	
Marital status	Single	556 (30.0)	111 (25.3)	0.140
	Married	1220 (65.8)	307 (69.9)	
	Divorced and widowed	77 (4.2)	21 (4.8)	
Self-described economic status	Low	216 (11.7)	61 (13.9)	0.416
	Medium	1451 (78.3)	337 (76.8)	
	High	186 (10.0)	41 (9.3)	
Self-described level of religious commitment	Low	50 (2.7)	6 (1.4)	0.005*
	Moderate	1198 (64.7)	257 (58.5)	
	High	605 (32.6)	176 (40.1)	
Region	Gulf	428 (23.1)	93 (21.2)	˂0.001**
	North Africa	98 (5.3)	33 (7.5)	
	Sub-Saharan Africa	112 (6.0)	99 (22.6)	
	Europe, North America, and others	21 (1.1)	4 (0.9)	
	Levant	1156 (62.4)	185 (42.1)	
	Asia	38 (2.1)	25 (5.7)	
Attitude total score	Good Attitude	1760 (95.0)	411 (93.6)	0.152
	Poor attitude	93 (5.0)	28 (6.4)	

* = *p *< 0.01, ** = *p *< 0.001

Similarly, females showed a more positive attitude toward the foods and remedies mentioned in the HQPS than males (*p* = 0.019). Participants who were employed had a more positive attitude than those who were unemployed or otherwise used (*p* = 0.046). Moreover, participants who described their religious commitment as moderate had a more positive attitude than those who reported high or low levels of religious commitment (*p* < 0.001). Additionally, participants from the Levant region exhibited a more positive attitude than those from the other areas (*p* = 0.014), as shown in Table 7.

**Table 7:** Distribution of sociodemographic characteristics based on the attitude level (*n* = 2,292)

Sociodemographic characteristic		Good attitude	Poor attitude	*P*-value
Sex	Male	841 (38.7)	59 (48.8)	0.019*
	Female	1330 (61.3)	62 (51.2)	
Educational level	less than a university	205 (9.4)	12 (9.9)	0.400
	University (BSc)	1244 (57.3)	62 (51.2)	
	Graduate (MSc/ PhD)	722 (33.3)	47 (38.8)	
Job/occupation	Housewife	321 (14.8)	11 (9.1)	0.046*
	Student	305 (14.0)	12 (9.9)	
	Worker/ Employee	1246 (57.4)	73 (60.3)	
	Retired / no work	299 (13.8)	25 (20.7)	
Marital status	Single	635 (29.2)	32 (26.4)	0.656
	Married	1442 (66.4)	85 (70.2)	
	Divorced and widowed	94 (4.3)	4 (3.3)	
Self-described economic status	Low	268 (12.3)	9 (7.4)	0.036*
	Medium	1695 (78.1)	93 (76.9)	
	High	208 (9.6)	19 (15.7)	
Self-described level of religious commitment	Low	46 (2.1)	10 (8.3)	˂0.001**
	Moderate	1375 (63.3)	80 (66.1)	
	High	750 (34.5)	31 (25.6)	
Region	Gulf	498 (22.9)	23 (19.0)	0.014*
	North Africa	123 (5.7)	8 (6.6)	
	Sub-Saharan Africa	199 (9.2)	12 (9.9)	
	Europe, North America, and others	20 (0.9)	5 (4.1)	
	Levant	1274 (58.7)	67 (55.4)	
	Asia	57 (2.6)	6 (5.0)	
Knowledge total score	Adequate knowledge	1760 (81.1)	93 (76.9)	0.152
	Inadequate knowledge	411 (18.9)	28 (23.1)	

* = *p *< 0.01, ** = *p *< 0.001

Furthermore, females exhibited more favorable practices regarding the foods and remedies mentioned in the HQPS than males (*p* < 0.001). Participants who were employed demonstrated better practices than those who were unemployed or otherwise used (*p* = 0.008). Moreover, participants who described their religious commitment as moderate demonstrated more favorable practices than participants who reported high or low levels of religious commitment (*p* < 0.001). Additionally, participants from the Levant region exhibited better practices than those from the other areas (*p* < 0.001). Participants with adequate knowledge demonstrated poor practice regarding foods and remedies mentioned in the HQPS (*p* < 0.001). In contrast, participants with a positive attitude towards foods and remedies mentioned in the HQPS demonstrated good practices (*p* = 0.002), as shown in Table 8.

**Table 8:** Distribution of sociodemographic, knowledge, and attitude characteristics based on the practice level (*n* = 2,292)

Sociodemographic characteristic		Good practice	Poor practice	*P*-value
Sex	Male	572 (43.7)	328 (33.4)	˂0.001**
	Female	738 (56.3)	654 (66.6)	
Educational level	less than a university	139 (10.6)	78 (7.9)	0.064
	University (BSc)	727 (55.5)	579 (59.0)	
	Graduate (MSc/ PhD)	444 (33.9)	325 (33.1)	
Job/occupation	Housewife	209 (16.0)	123 (12.5)	0.008*
	Student	159 (12.1)	158 (16.1)	
	Worker/ Employee	749 (57.2)	570 (58.0)	
	Retired / no work	193 (14.7)	131 (13.3)	
Marital status	Single	315 (24.0)	352 (35.8)	˂0.001**
	Married	931 (71.1)	596 (60.7)	
	Divorced and widowed	64 (4.9)	34 (3.5)	
Self-described economic status	Low	153 (11.7)	124 (12.6)	0.214
	Medium	1038 (79.2)	750 (76.4)	
	High	119 (9.1)	108 (11.0)	
Self-described level of religious commitment	Low	17 (1.3)	39 (4.0)	˂0.001**
	Moderate	771 (58.9)	684 (69.7)	
	High	522 (39.8)	259 (26.4)	
Region	Gulf	282 (21.5)	239 (24.3)	˂0.001**
	North Africa	76 (5.8)	55 (5.6)	
	Sub-Saharan Africa	155 (11.8)	56 (5.7)	
	Europe, North America, and others	15 (1.1)	10 (1.0)	
	Levant	740 (56.5)	601 (61.2)	
	Asia	42 (3.2)	21 (2.1)	
Knowledge total score	Adequate knowledge	995 (76.0)	858 (87.4)	˂0.001**
	Inadequate knowledge	315 (24.0)	124 (12.6)	
Attitude total score	Positive Attitude	1258 (96.0)	913 (93.0)	0.002**
	Negative attitude	52 (4.0)	69 (7.0)	

* = *p *< 0.01, ** = *p *< 0.001

### Structural model

**Fig. 5:**
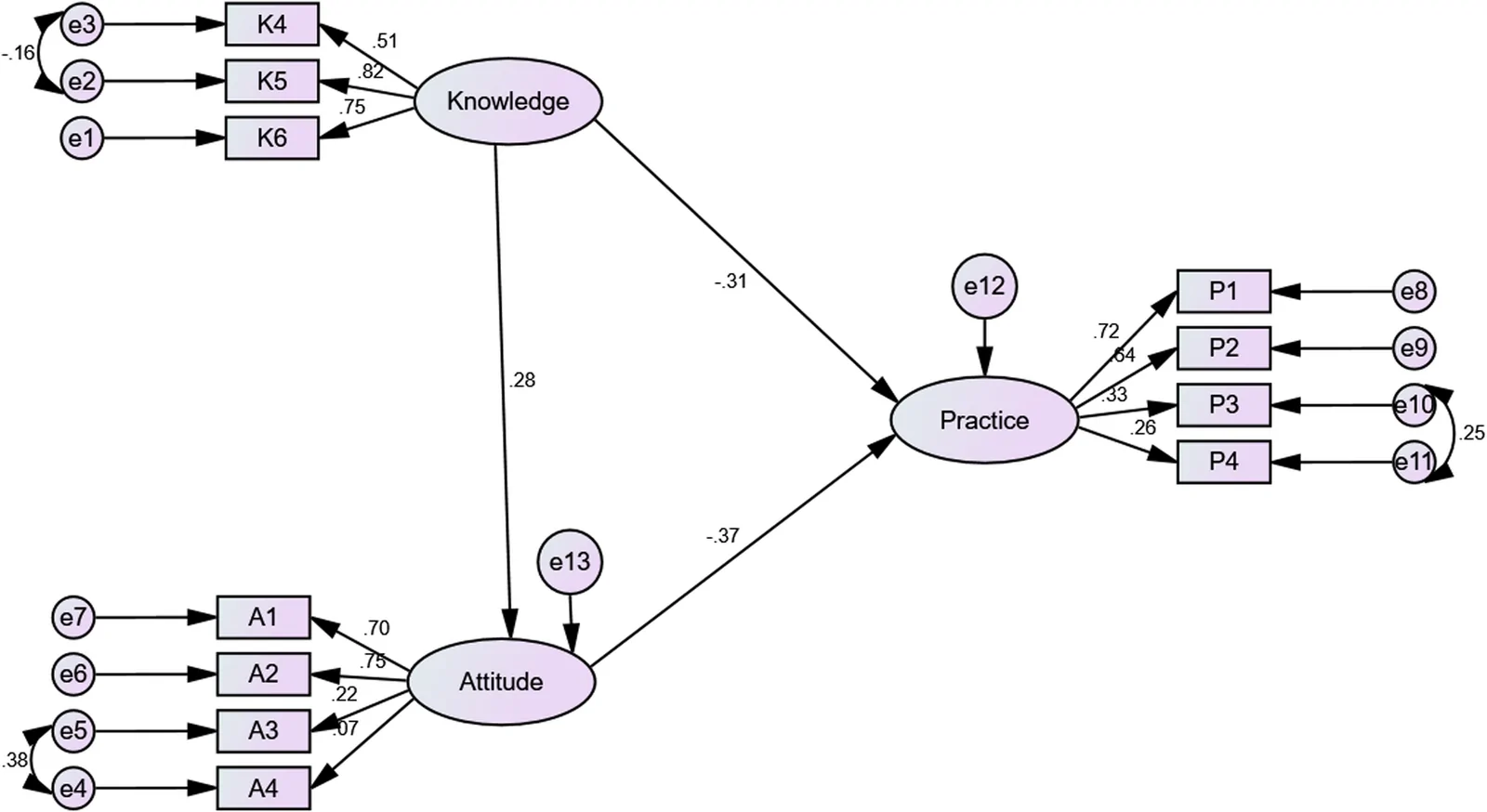
The relationships test results of knowledge, attitude, and practice (*n* = 2,292)

A structural model (Fig. 5) was developed to examine the relationships between knowledge, attitudes, religious commitment, and practices, as proposed in Fig. 1. Structural Equation Modelling (SEM) testing results indicate that the proposed relationships are supported, as shown in Table 9. Hence, each knowledge and attitude has a significant negative influence on practices. Knowledge has a stronger impact on practices than attitude. Furthermore, Knowledge has a positive effect on attitude. The results show that the goodness-of-fit indices presented in Table 10 were within the acceptable limits. The goodness-of-fit indices for a structural model are as follows: The chi-square to degrees of freedom ratio (χ²/df) = 5, Root Mean Square Residual (RMR) = 0.044, and the values of Comparative Fit Index (CFI), Incremental Fit Index (IFI), and Tucker-Lewis Index (TLI) were 0.956, 0.956, and 0.956, respectively. Lastly, the Root Mean Square Error of Approximation (RMSEA) = 0.047. Taken together, these indices indicate that the structural model achieved an acceptable and adequate level of fit.

**Table 9:** The relationships test results of knowledge, attitude, and practice (*n* = 2,292)

	Relationships	Standardized estimate	Standard error	Critical ratio	*P*-Value
1	Knowledge to Attitude	0.029	0.011	2.695	0.007
2	Knowledge to Practice	− 0.299	0.033	-8.929	0.000
3	Attitude to Practice	-3.446	1.252	-2.753	0.006

**Table 10:** Goodness of fit indices for structural model (*n* = 2,292)

Fit indices	Model value	Accepted value	Reference
χ2/df	5	χ2/df ≤ 5	Schumacker and Lomax (2004)
RMR	0.044	RMR ≤ 0.08	Browne and Cudeck (1993)
CFI	0.956	> 0.90	Byrne (2010)
TLI	0.936	> 0.90	Byrne (2013)
IFI	0.956	> 0.90	Byrne (2013)
RMSEA	0.047	< 0.10	Byrne (2013)

**Fig. 6:**
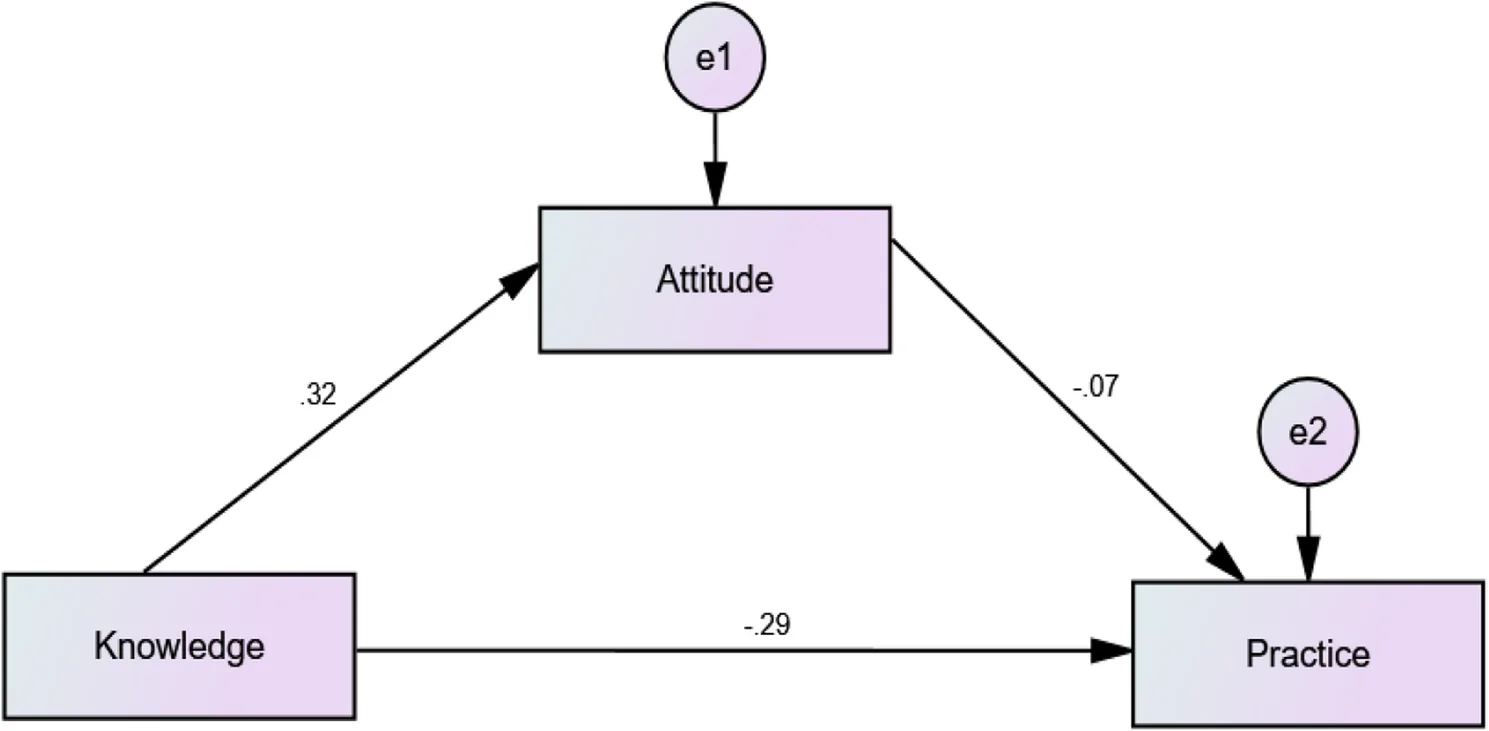
Mediation tests. (*n* = 2,292)

**Fig. 7:**
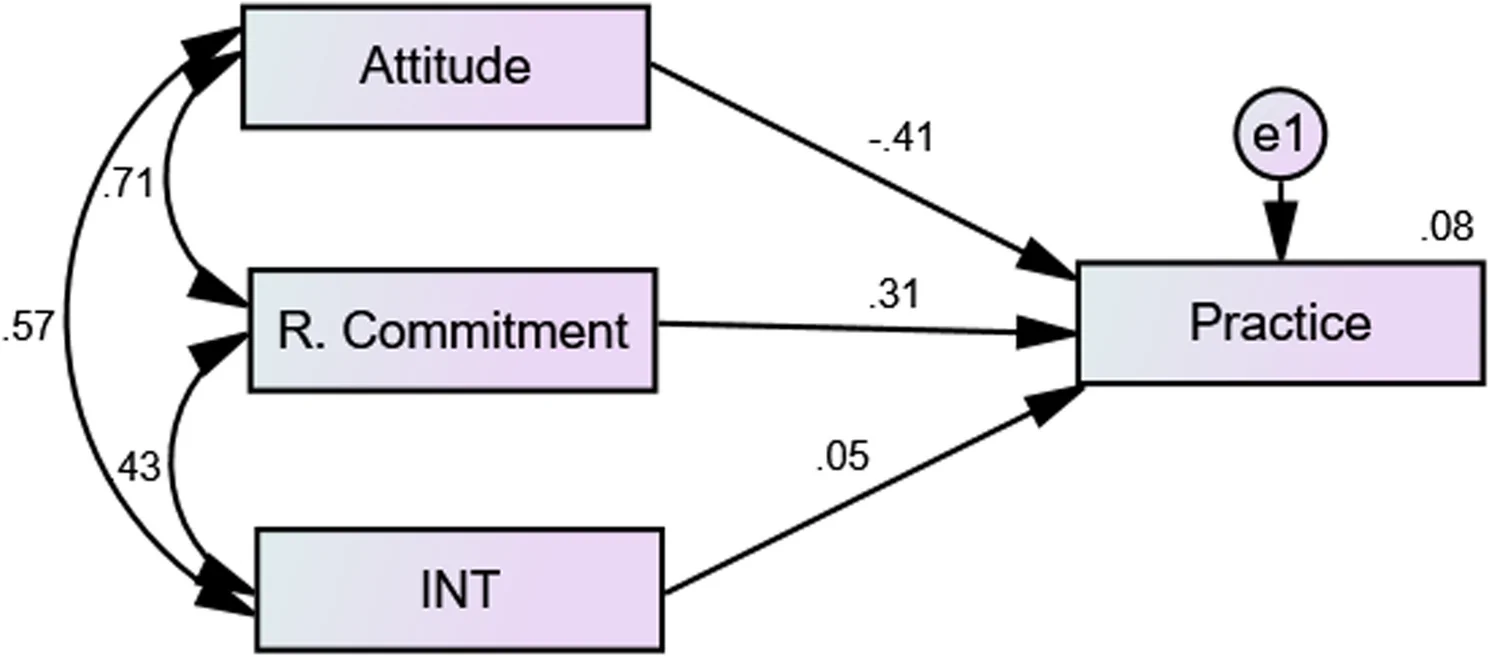
The negative direct effect of attitude on practice, the positive direct effect of religious commitment, and the moderation effect of religious commitment bridge the gap. (*n* = 2,292)

Figure 6 shows that attitude acts as a partial mediator in the relationship, both directly (*P* < 0.001) and indirectly (*P* < 0.01), as presented in Table 11. Figure 7 presents the results of the moderation analysis in the KAP model, examining the role of religious commitment in moderating the relationship between attitude and practice. As shown in Table 11, this factor had a positive and significant influence on practices (Estimate = 0.309, *p* < 0.001). Furthermore, the interaction term (attitude × religious commitment) shows a positive, statistically significant effect (Estimate = 0.030, *p* = 0.026). The negative direct effect of attitude on practice, the positive direct effect of religious commitment, and the moderating effect of religious commitment bridge the gap (Table 12).

**Table 11:** Mediation tests (*n* = 2,292)

Relationship	Mediator	Standardized direct effect	Standardized indirect effect	Mediation Result
Knowledge to Practice	Attitude	− 0.292***	− 0.021**	Partial

** = *p* < 0.01, *** = *p* < 0.001

**Table 12:** The negative direct effect of attitude on practice, the positive direct effect of religious commitment, and the moderating effect of religious commitment bridge the gap (*n* = 2,292)

	Path	Standardized estimate	Standard error	Critical ratio	*P*-Value
1	Attitude to Practice	-0.409	0.031	-13.074	***
2	Religious Commitment to Practice	0.309	0.028	10.885	***
3	Attitude*Religious Commitment to Practice	0.030	0.014	2.231	0.026

## Discussion

KAP studies are among the most important types of studies for identifying levels of knowledge, trends, and behaviors related to health, nutrition, and life phenomena across various populations [50]. Our study helps bridge the gap between cultural beliefs and modern healthcare practices. To our knowledge, this is the first study of its kind attempting to explore the KAP among adult Muslims concerned with foods and remedies mentioned in the HQPS in the context of modern medical treatments. Multiple studies have indicated the potential benefits and mechanisms of action of foods and remedies mentioned in Islamic heritage in treating chronic and acute diseases, with particular emphasis on those in the HQPS, which hold significant religious significance [7, 42]. In the present study, participants demonstrated increased awareness of the importance of sound scientific principles in using food and natural remedies to protect the body from deterioration and harm. Moreover, there was a strong belief in the use of foods (such as honey, garlic, and black seed) and treatments (such as Hijama and cauterization) mentioned in the HQPS as aids to traditional therapies for treating and preventing disease. This strong belief among participants aligns with the focus of contemporary research on the health benefits and medicinal value of the foods mentioned above. Modern scientific literature suggests plausible biological mechanisms for the health benefits and medicinal value of the foods mentioned above due to their abundance of micronutrients and natural bioactive compounds, such as antioxidants, anti-inflammatory, and anti-carcinogenic compounds [8, 9, 18, 19, 41, 51].

When participants were asked whether the foods and remedies mentioned in the HQPS could serve as effective alternatives to traditional or modern medical treatments, their responses varied widely. Differences in opinion can be attributed to varying levels of education, experience, and awareness within the health and medical fields. It can also be attributed to cultural and societal differences in how people approach these foods and remedies, as well as their attitudes toward them. Variations in educational level, race, and culture are considered influential factors that affect health awareness levels in contemporary societies [52]. Hence, targeted educational programs could bridge the gaps by integrating religious and scientific perspectives.

The differences and discrepancies in respondents’ opinions regarding the use of foods and remedies mentioned in the Prophetic Sunnah in medical therapy may also be attributed to divisions among Islamic jurisprudential schools. Contemporary Muslim scholars have adopted varying standpoints, as noted by Al-Ashqar in his Doctoral dissertation titled: *“The Actions of the Messenger (peace be upon Him) and its Implications for Islamic Ruling.”* [53] and in his article titled: “*The Authority of the Prophetic Sunnah in the Field of Medicine.”* [54]. Scholars hold differing views on the Prophet’s sayings and actions regarding medical therapy. Some believe these reflect his human nature and personal experiences and therefore are non-legislative. Others argue that these sayings and actions should be treated as religious legislation. The latter group supports their stance based on the Qur’anic verse regarding the Prophet *(Nor does he speak out of (his own) desire ‘3’. This is nothing but an inspired revelation ‘4’)*,* in Chap. 53*,* verse 3–4*. This is supported by Zamoul in his book “The Authority of Hadiths in the Prophetic Sunnah on Medicine and Treatment.” [55] and by Dr. Waseem Da’na in his doctoral dissertation on “Prophetic Medical Treatments in Light of Modern Pharmacological Studies: A Comparative Doctrinal Study.” [49].

The first group, however, differentiated between the Prophet’s sayings and actions as a human being influenced by the physical and intellectual environment and as a legislating Prophet, referring to events like the pollination of date palms, the wells of Badr, and treatments, including Indian Costus, cupping therapy, and cauterization, which were common during the Prophetic era [56]. Some scholars have even suggested that the Prophet and his companions sought medical advice and treatment beyond the Prophet’s practices. For instance, his companion Sa’d ibn Abi Waqqas reported that when he fell ill, the Prophet visited him and said: *“You are suffering from a heart condition—go to Al-Harith ibn Kalda*,* the brother of Thaqeef*,* for he practices medicine”* [57].

Muslim scholars concluded that *Sharia* prioritizes protecting human health over Prophetic *Sunnah* practices in the medical context, which may often reflect the Prophet’s human experience rather than legislative intent [58]. In the same context, there are differing perspectives on how to approach the use of foods and treatments mentioned in the HQPS in healthcare. Some scholars believe that it is crucial to adhere strictly to the Prophetic guidance as an act of worship and a means of following the Prophet’s example. However, other scholars emphasize the importance of integrating these traditional practices with modern medicine. They advocate the use of HQPS foods and remedies as complementary therapies within a comprehensive treatment plan, emphasizing the need for evidence-based practices to ensure their safety and efficacy. Ultimately, the two perspectives can be reconciled by recognizing the value of traditional and modern approaches to healthcare. Adhering to Prophetic guidance on foods and remedies can be considered within the framework of holistic medicine, provided that evidence-based practices inform their use and that they complement modern medical treatments. It is crucial to ensure that any traditional remedy is safe and effective and does not interfere with or contradict established medical treatments. Thorough research and clinical evidence are essential for validating the safety and efficacy of any traditional remedy before it is widely used.

Participants’ responses toward their beliefs about whether foods and remedies mentioned in the HQPS cause no harm regardless of the quantity, method of approach, or medical conditions of the person reflected high levels of knowledge and health practices. It is a misconception to believe that using certain foods or remedies, such as honey or palm dates mentioned in the HQPS, is universally safe for everyone, regardless of health status. For example, honey or dates can harm a diabetic patient if consumed in excessive amounts, and cupping therapy may be harmful to those with heart disease or vascular issues, such as myocardial infarction [59]. The effectiveness and safety of such treatments must be considered with the individual’s specific health condition [13, 60].

When examining the sources from which participants obtained their information about foods and remedies mentioned in the HQPS, surprisingly, most participants relied on religious books, scientific journals, and specialized professionals such as doctors, nutritionists, and alternative medicine practitioners, keeping in mind that some alternative medicine specialists are not qualified in medical, health, or nutritional sciences. This is a positive sign, consistent with the fact that most participants are scientifically qualified and hold undergraduate and/or graduate degrees, given the connection between foods and remedies in the fields of health, medicine, and nutrition.

Moreover, most participants supported raising awareness of foods and remedies mentioned in the HQPS as complementary therapeutics and part of holistic medicine. Advocating for their inclusion in educational curricula for younger generations, participants also emphasized the need for regulatory oversight and professional monitoring to ensure these practices are used safely and effectively under medical and governmental supervision. This reflects a high level of health awareness of the risks associated with improper use of HQPS foods and medicines, including the potential for poisoning, infection, and the transmission of infectious diseases. Past incidents include infections linked to improperly sterilized cupping tools [61] and harmful practices involving camel urine [62]. This matter was also discussed in a previous review article on CAM use within the Protestant Christian Church, which emphasized the importance of regulating CAM in North America to guide church congregants [63]. Overall, participants had a highly positive attitude toward the foods and remedies mentioned in the HQPS. This attitude was positively correlated with their high knowledge scores.

When considering the regular practices of consuming foods mentioned in the HQPS for prevention, healing from disease, or maintaining health, responses align with participants’ high level of religious commitment and their belief in food’s role in preserving health, protecting the body, and preventing harm. The frequent use of olive oil, honey, and palm dates (especially Ajwa dates) and *Zamzam* water suggests an increased awareness of their health benefits, including their anti-inflammatory, antioxidant, cancer-fighting, and immune-boosting properties [41, 64, 65]. Therefore, community health programs could leverage the influence of religious leaders to promote safe practices that align with Islamic teachings.

The most commonly used therapies were fasting, water therapy to reduce fever, and cupping therapy. Fasting, present in the form of religious intermittent fasting, is a common practice among Muslims and is gaining significant attention as a health-promoting strategy. Emerging evidence highlights the physiological benefits of intermittent fasting, including improvements in metabolic health, weight management, reduced inflammation, and enhanced cellular repair processes such as autophagy [66]. This renewed interest has spurred a growing body of research on the mechanisms, benefits, and applications of fasting in clinical and non-clinical settings [67–69]. Regarding cupping therapy, multiple recent clinical studies have come to show some beneficial effects, such as activating the immune system and treating certain diseases such as fibromyalgia, fibrosis, cervical spondylosis, sciatica, gout, arthritis, skin conditions, pain, hyperlipidemia, persistent low back pain, chronic non-specific neck pain, non-specific lower back pain, acute and chronic pain management, migraines, cellulitis, and gynecological disorders [11, 12, 70].

However, the practice of cauterization and camel urine consumption was the least common, which can be attributed to the associated pain, potential health risks, and the ongoing religious debates, especially regarding camel urine consumption [71]. Moreover, most participants reported striving to follow the Qur’anic guidance and prophetic tradition to avoid food waste, reflecting their moderate to high levels of religious commitment and high levels of education. Reducing food waste is linked to moderation in food intake [72], which is positively associated with improved awareness of the risks of overeating, such as obesity [73]; the main risk factor for diabetes, heart disease, stroke, cancer, joint diseases, and other chronic illnesses [74].

The majority of participants’ commitment to food and drink etiquette, such as washing hands before and after meals, also reflects their high to moderate level of religious adherence and further indicates an understanding of the importance of this practice in preventing illnesses caused by microbial contamination of food, such as food poisoning [75, 76]. Finally, the participants’ insistence on verifying the authenticity of information related to foods and remedies mentioned in the HQPS aligns with their high level of education and adherence to the religious methodology in confirming and verifying information, rather than simply accepting what is spread by word of mouth or on social media [77]. Participants who possessed adequate knowledge and exhibited a positive attitude and practices were most likely to be employed females with a bachelor’s degree. Adequate knowledge, a positive attitude, and practices were also more common among participants from the Levant who described themselves as moderately committed to their religion. However, participants with adequate knowledge were more likely to exhibit unhealthy practices.

The relationship between KAP and health concerns is complex and multifaceted. While adequate knowledge can significantly enhance attitudes toward health practices, it does not always translate into actual improvements in health behaviors. This phenomenon can be attributed to several factors, including socioeconomic conditions, psychological barriers, and the complexity of behavior change. The current study revealed a positive correlation between adequate knowledge, a positive attitude, and good health practices. However, it also showed a negative correlation between adequate knowledge and good practices. These results align with numerous KAP studies, which show that while individuals may possess adequate understanding of health-related topics, this knowledge does not necessarily translate into improved practices [78–80]. It is conceivable that fostering positive attitudes within communities—such as incorporating foods and remedies mentioned in the HQPS into school curricula, providing healthcare professionals with proper training on integrating these foods and remedies into modern medical practices, and ensuring effective communication with patients and the broader community—could lead to a significant improvement in health practices. Additionally, enforcing government regulations, such as requiring valid certification for cupping therapists or ensuring quality control of herbal products, could enhance safety and efficacy, thereby further supporting the safe implementation of these practices.

According to earlier studies employing the KAP model, knowledge cultivates positive attitudes, which in turn impact promising practices [34]. Findings about how these elements relate to one another, however, have been mixed. In this study, both knowledge and attitude affect practices, with knowledge having a greater effect. Contrary to certain studies [35], which found that knowledge had no discernible effect on practices; knowledge also has a considerable impact on attitudes [36].

Consistent with the findings of Baser et al., the present study also finds that attitude partially mediates the relationship between knowledge and practice [37]. The negative relationship between attitudes and practices, however, suggests that attitudes alone do not always result in consistent practices. This could be due to overconfidence or external factors such as organizational hurdles, insufficient resources, or lack of motivation. Religious commitment, introduced as a moderator, is significantly positively associated with practices. The interaction between attitude and religious commitment highlights its role in bridging the attitude-practices gap, encouraging moral accountability and action drive.

This underscores the value of religious commitment [38] and ethical frameworks, such as Al-Ehssan, in fostering consistent, values-driven behaviors [36]. Variations in respondents’ attitudes, a critical component in converting knowledge into practices, may be reflected in differences across studies’ results [81]. These findings underscore the importance of identifying and accounting for influencing factors, such as religious commitment, to ensure that attitudes and knowledge are effectively translated into practice.

### Strengths and limitations

As the first of its kind in the available literature, the current study exhibits several strengths. First, it offers the most comprehensive coverage of all foods and remedies mentioned in the HQPS. Second, it holds the largest sample size among related studies. Additionally, the study covers the broadest geographical scope, encompassing multiple regions. However, this study entails some limitations that must be considered when interpreting its findings. Although the study included a large sample, participants were recruited through convenience and snowball sampling, which limited the generalizability of the findings. Recall bias in some questions can lead to inaccuracies. The selection bias resulting from the restricted recruitment of individuals familiar with or who use electronic devices and social media platforms is another limitation to consider. The reliance on online and social media recruitment may also have led to selection bias, as individuals without internet access or unfamiliar with social media platforms were less likely to participate, potentially affecting the representativeness of the sample. The lack of generalizability of the current findings to all adult Muslims worldwide, due to the convenience sampling employed and the limited number of Islamic countries involved, is another limitation. Social desirability bias may have influenced participants to report behaviors or beliefs perceived as favorable. Nonresponse bias is also possible, since not all individuals invited to participate completed the questionnaire. The inherent limitation of the observational design, due to the coexistence of unmeasured factors, may also have influenced participants’ awareness and practices. Additionally, the cross-sectional design precludes inferring directionality or causality between variables. Still, these were not captured in the study for various reasons.

## Conclusion

The study highlights the association between the foods and treatments mentioned in the HQPS on health KAP among adult Muslims, noting strong beliefs in their health benefits, often supported by modern scientific evidence. It is necessary to approach HQPS from a comprehensive medical perspective that integrates contemporary medical knowledge with an understanding of HQPS texts, thereby minimizing potential risks, complications, and adverse interactions, particularly when medical conditions may conflict with its use. Key recommendations include promoting educational initiatives to enhance awareness of their proper use, based on *Sharia* understanding regarding these foods and treatments, to emphasize their use by what is stated in the HQPS texts and under the supervision of medical specialists, and to work on establishing laws and legislation regulating the medical practices mentioned in the HQPS, especially those related to cupping, cauterization, camel urine, and others, in a way that achieves the interest of the patient and combines the rules of *Sharia* in preserving the soul and body with the outcomes of modern science, and in a way that achieves the interest of the Muslim and humanity as a whole, and to see the state of conflict between those practices related to the foods and treatments mentioned in the HQPS on the one hand, and the correct understanding of the *Sharia* texts. The study also recommends conducting further research on the foods and remedies mentioned in the HQPS to determine the identities (compositions) of the active ingredients, the effective doses, and the optimal methods of use. The study also emphasizes the importance of integrating traditional religious practices with evidence-based medicine through culture-centered training for healthcare providers and implementing regulatory oversight for practices such as cupping therapy to support patient safety and well-being. A balanced approach that integrates cultural and religious values with scientific rigor can improve health outcomes and support holistic patient care.

## Data Availability

Data are available from the authors upon request.

## Abbreviations

CFI: Comparative Fit Index
CAM: Complementary and alternative medicine
HQPS: Holy Qur'an and the Prophetic Sunnah
IFI: Incremental Fit Index
KAP: Knowledge, attitudes, and practices
RMR: Mean Square Residual
RMSEA: Root Mean Square Error of Approximation
SD: Standard deviation
SEM: Structural Equation Modelling
TAIM: Traditional Arabic & Islamic Medicine model
TLI: Tucker-Lewis Index
